# The Association between Chronic Arsenic Exposure and Hypertension: A Meta-Analysis

**DOI:** 10.1155/2012/198793

**Published:** 2012-03-08

**Authors:** Tanvir Abir, Bayzidur Rahman, Catherine D'Este, Abdulaziz Farooq, Abul Hasnat Milton

**Affiliations:** ^1^Centre for Clinical Epidemiology and Biostatistics (CCEB), The School of Medicine and Public Health, Faculty of Health, The University of Newcastle, Newcastle, NSW 2008, Australia; ^2^The School of Public Health and Community Medicine, Faculty of Medicine, The University of New South Wales, Sydney, NSW 2052, Australia; ^3^Biostatistics, School of Medicine and Public Health, The University of Newcastle, Newcastle, NSW 2008, Australia; ^4^Research and Education Department, ASPETAR-Qatar Orthopaedic and Sports Medicine Hospital, P.O. Box 29992, Doha, Qatar

## Abstract

*Background*. There is inconclusive evidence from cross-sectional and cohort studies that arsenic exposure is a risk factor involved in the development of hypertension. *Methods*. A database search, using several keywords, was conducted to identify relevant studies. Separate odds ratio estimates for arsenic exposure with concentration only and arsenic exposure with duration, including biomarker, were extracted from studies that met all inclusion criteria. The extracted odds ratios (OR) comparing the highest exposure categories with the lowest in each study were pooled using the random effects methods of meta-analysis. Heterogeneity of odds ratios in the included studies were analyzed using I^2^ statistics. *Results*. Eight studies were analyzed. Using the exposure as arsenic concentration in the drinking water, the OR estimate was 1.9 (95% CI: 1.2–3.0), with the I^2^ = 92%, while using the exposure as concentration and duration, the OR estimate was 1.4 (95% CI: 0.95–2.0) with the I^2^ = 80%. Meta-regression was done and the quality of exposure measurement was found to be significantly associated with the effect measure. For a one unit increase in the score from exposure assessment, the odds ratio decreased by 6%. No publication bias was evident. The only major weaknesses of this study were heterogeneity across studies and small sample size. *Conclusions*. The study findings provide limited evidence for a relationship between arsenic and hypertension. In summary, the relationship between arsenic exposure and hypertension is still inconclusive and needs further validation through prospective cohort studies.

## 1. Introduction

Arsenic is a known carcinogen [1], and increasing evidence also suggests a possible role of chronic arsenic exposure in the development of chronic diseases, including cardiovascular diseases [2]. Cardiovascular diseases associated with high levels of arsenic in the drinking water include atherosclerosis, hypertension, cerebrovascular diseases, ischemic heart diseases, and peripheral vascular disease [3, 4].

The possible role of chronic arsenic exposure in the development of hypertension has been previously reported from Taiwan, Bangladesh, and the United States [5–7]. These studies reported that individuals drinking arsenic-contaminated water for a long period of time might be at higher risk of developing hypertension compared to those unexposed to arsenic, and the effects were dose dependent.

Hypertension is one of the major risk factors for cardiovascular diseases [8] and is considered a major public health problem worldwide [9]. A total of 972 million adults were reported to have hypertension in 2000 and that number is estimated to increase by 60%, to a total of 1.56 billion, by the year 2025 [9]. The economic costs associated with hypertension are also very high.

To date, only a few cross-sectional and cohort studies have investigated the association between hypertension and chronic arsenic exposure [10]. Therefore, it is important from the public health perspective to explore the role of chronic arsenic exposure in development and prevention of hypertension. In this study, we conducted a meta-analysis that included all relevant cross-sectional and cohort studies of chronic arsenic exposure and hypertension. The meta-analysis provides greater precision in estimating risk especially when data are scarce and inconclusive. Our analysis includes a careful assessment of the possible effects of study quality as well as other biases on the meta-analysis results.

## 2. Materials and Methods

### 2.1. Literature Search

We used a comprehensive search strategy to identify all relevant studies. The research question was defined as “Is there any association between chronic arsenic exposure and hypertension?” This question was then broken down to cover specific search terms such as “hypertension,” “chronic arsenic exposure,” “arsenite,” “arsenosis,” “arsenicosis,” “diastolic,” and “systolic.” Each term was included in one or more searches. The search was first carried out in Ovid MEDLINE and Google Scholar in September 2009, without limitation on the time of publication. We also replicated the search in EMBASE and PubMed, but no additional references were identified through these databases. The search was last updated in February 2012 to locate new studies published after the initial search. No new studies on this topic had been published. The references cited in the collected list of articles were also hand-searched for additional relevant studies.

### 2.2. Selection of Studies

The included studies (1) were observational studies, that is, cross-sectional, case control or cohort studies, of chronic arsenic exposure through drinking water and hypertension, (2) reported a relative risk (RR) or odds ratio (OR), or such parameters could be estimated from the published data, and (3) measured arsenic, using a laboratory technique.

Two investigators (T. A. and R. E. Y.) screened the initial list of articles to identify articles that were irrelevant and, therefore, discarded from the list. To facilitate faster screening, articles were first judged on the basis of their titles, where obviously irrelevant articles were discarded. The abstracts of the remaining articles were then read by three investigators (T. A., B. R., and A. H. M.) to ensure that the main subject of the study was related to arsenic exposure and hypertension. Articles whose abstracts showed that the study was not relevant to the objective of the meta-analysis were discarded. Final screening of the articles was based on the full content. After the screening, information for the articles was extracted and a quality evaluation was done.

### 2.3. Data Extraction

A data extraction format was prepared to extract relevant information from the individual studies. The data items extracted from each study were

- general information: first author's name and affiliation, year of publication, country, and region in which the study was conducted;
- study design: population, number and sources of study participants, and type of study;
- exposure and outcome measurement: analytical methods for measuring arsenic, speciation of arsenic, duration of arsenic exposure, relevant information on individual level of exposure assessment, and measurement of hypertension;
- analysis: covariates for adjustment in multivariate models, adjusted and unadjusted or stratified effect estimates (i.e., RR and OR) and 95% confidence intervals (CIs). If a study did not report CIs, the standard error and the resulting CI from the *P* value were estimated [11], where possible.

For cross-sectional studies, ORs were extracted as the estimate of the effect of arsenic exposure. For cohort studies, the RRs were used.

### 2.4. Quality of the Studies

Each article was evaluated in a standardised manner to assess its individual qualities. Quality assessment was facilitated through the use of a standardised questionnaire which was developed specifically for this study. The questionnaire was a comprehensive scoring instrument based on critical appraisal principles and understanding of the methodological issues of population selection, measurement of arsenic exposure and outcome, adjustment for potential confounders, and appropriateness of analysis. Each criterion was given weighted scores to evaluate the quality of the studies in comparison to each other, with key factors being given specific scores. Although the scoring in itself can be subjective, it enables comparisons among the studies. Three independent readers (T. A., R. E. Y., and A. F.) scored each of the studies and were blinded to the others' assessments. Any significant disagreement between the readers was resolved through discussion. For minor differences, the average score was used as the agreed score. This resulted in a single agreed score for each characteristic of each paper.

The maximum score value that could be assigned to each study was 100, and this score summarised the overall quality of the study. The score was the weighted sum of four major domains of quality: selection issues (score weight 20%), measurement issues (of exposure and outcome, score weight 50%), adjustment of confounding factors (score weight 20%) and analysis (score weight 10%). The analysis section was given the least weight because if the other three sections were of good quality but the analysis was not carried out properly, the data could often be reanalyzed. Two scoring systems were developed, one for cross-sectional studies and one for prospective studies. Each of the studies was subjectively assessed and scored using these instruments.

## 3. Statistical Analysis

To assess the interrater reliability of the study quality scores, reliability and reproducibility were analyzed by computing intraclass correlation coefficients (ICCs) for absolute agreement between raters on each quality domain along with the overall score.

To conduct the meta-analysis, ORs were extracted from cohort studies and used to estimate the effects, with the exception of one cohort study where the RR was used instead. For studies where several measures of association were reported, associations which related hypertension to amount of arsenic intake, or to arsenic intake combined with duration of exposure, were considered. Pooled ORs were estimated by comparing the highest exposure category to the lowest one combined for males and females.

The meta-analysis included checking for heterogeneity of the ORs, checking for influential studies, checking for asymmetry and publication biases, and checking for relationships between the log of effect measures and the article's quality scores.

Effect estimates (ORs) were pooled using DerSimonian and Laird's inverse-variance-weighted random effects method [12]. Heterogeneity was measured using the I^2^-statistic [13], which describes the proportion of total variation in study estimates that is due to heterogeneity [13]. The influence of each study on the pooled effect measure was examined by repeating the meta-analysis while omitting each study one by one.

A Funnel plot was used to check for potential publication bias [14]. This was also objectively measured using Begg's statistic [15]. A random effect metaregression was conducted using the logs of the effect measures (OR) as the outcome variable and the study quality score as the explanatory variable to assess the possible impact of study quality on the effect measures. These random effect models were fitted with two additive variance components: within study variance and between study residuals [16, 17]. All meta-analysis was conducted using STATA version 11.0 SE.

## 4. Results

### 4.1. Literature Survey

The database search yielded 85 articles, 10 of which investigated the association between chronic arsenic exposure and hypertension. Two of these articles were excluded from the meta-analysis because the full texts were not available. Thus, the final meta-analysis comprised eight studies: seven analytical cross-sectional studies and one prospective cohort study. Of these eight studies, two reported arsenic concentration only as exposure, two reported arsenic concentration and duration of drinking arsenic-contaminated water as a combined exposure value, and three reported arsenic concentration and arsenic concentration and duration of exposure separately.  Table 1 provides a summary of the relevant studies.

### 4.2. Quality Scoring

The average total scores and average per criterion scores for quality assessment are summarized in Table 2. The scores are percentages out of the highest possible score per criterion. The one cohort study scored better in the domains of *Selection *and *Outcome* compared to the other seven cross-sectional studies. The cohort study had a *Selection* score of 18.7 (of 20 possible), while the cross-sectional studies averaged 15.8. The cohort had an *Outcome* score of 16 (of 20 possible), while the 7 cross-sectional studies averaged 14.7. In particular, the study by Zierold et al. [18] scored low in this domain (1.3) due to missing details in the report. The 7 cross-sectional studies scored higher in the *Exposure* (cross-sectional = 24.3, cohort = 20, of 30 possible) and *Confounders *(cross-sectional = 18.6, cohort = 10, of 20 possible) domains. The *Analysis *(cohort = 9.3, cross-sectional = 10, of 10 possible) domain scores were similar; but, overall, the cross-sectional studies (total score = 84.1) scored higher than the cohort study (total score = 74). Good interobserver agreement on the total quality scores was verified by the intraclass correlation coefficient (ICC). The total score ICC was 0.88 (95% CI: 0.62–0.98). The highest agreement was seen among scores for the *Exposure *(ICC = 0.87 95% CI:  .56–.98) and *Outcome *(ICC = 0.96 95% CI: 0.64–1.00) domains, and the lowest for the *Analysis* (ICC = 0.001 95% CI: −0.24–0.78) domain. The low ICC, however, was due to one rater's disagreement on one study, but there was perfect agreement among the raters on the rest of the studies. Other domains showed moderate agreement: the ICC for *Confounders *was 0.59 (95% CI: −0.19–0.90) while that for *Selection* was 0.66 (95% CI: −0.01–0.93).

The eight selected studies were scored based on criteria that were specifically developed to assess their quality. The cohort study by Wang et al. [5] scored a total of 74 points, having scored well on *Selection* and *Exposure* but not on *Confounder* criteria. The seven cross-sectional studies had an average score of 84.1 (range = 61 to 93.3). Overall, the cross-sectional studies scored higher than the prospective cohort study for the *Confounders* and *Exposure* criteria but scored lower for the *Selection* and *Outcome* criteria. The summary of scores is presented in Table 2.

### 4.3. Adjustment of Potential Confounders

The studies differed in the adjustment of potential confounders, such as age, sex, and body mass index, as well. Such differences may add to the heterogeneity of the conclusions presented in the articles.

## 5. Risk Estimate and Meta-Analysis

**Figure 1:**
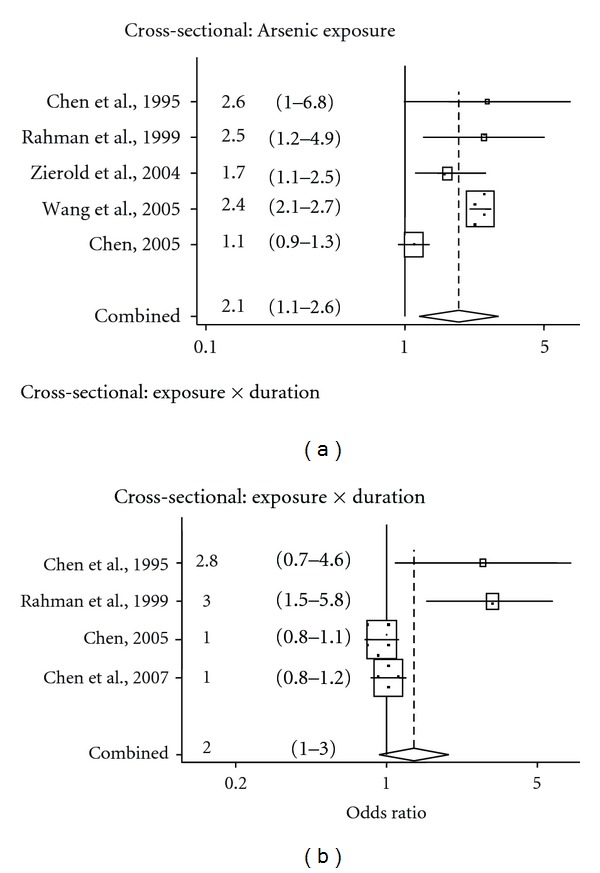
Forest plot of meta-analysis of arsenic and hypertension in cohort and cross-sectional studies.

The pooled OR of studies using arsenic concentration as the exposure measure was 1.9 (95% CI: 1.2–3.0), while the pooled estimate for OR of studies using arsenic concentration and duration including biomarker as the exposure was 1.4 (95% CI: 0.95–2.0). For the arsenic concentration studies, the I^2^ was 92% (*P* < 0.001), while, for the concentration and duration studies, I^2^ was 80% (*P* < 0.001). A *P* value <0.001 suggests a strong heterogeneity across studies. The meta-analysis results are presented with forest plots in Figure 1. In the forest plots, the point estimate for one study is represented by the centre of each rectangle. The size of the rectangle is relative to the weight of the point estimate when the results are pooled. The length of the horizontal line through the rectangle represents the 95% confidence interval of the point estimate. The pooled estimate is represented by the diamond on the lower portion of the plot. The vertical axis through the diamond represents the point estimate, and the horizontal end corners are the upper and lower 95% confidence boundaries.

### 5.1. Influence Analysis

**Figure 2:**
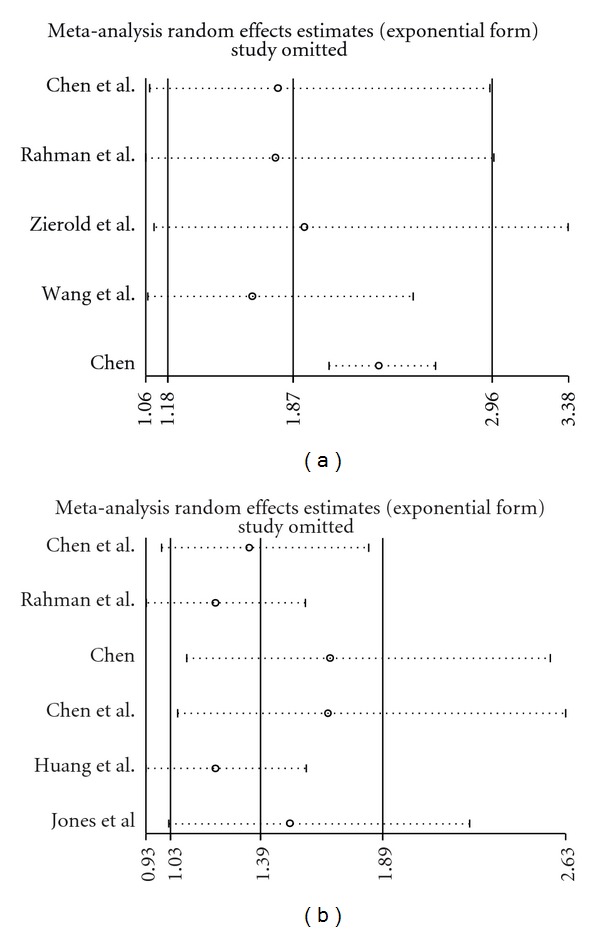
(a) Influence analysis for arsenic concentration as exposure assessment, (b) influence analysis for arsenic concentration and duration as exposure assessment.

The results of the influence analysis showed that no single study exerted a disproportionate influence on the pooled estimate for either arsenic concentration or arsenic concentration and duration (Figure 2).

### 5.2. Publication Bias

**Figure 3:**
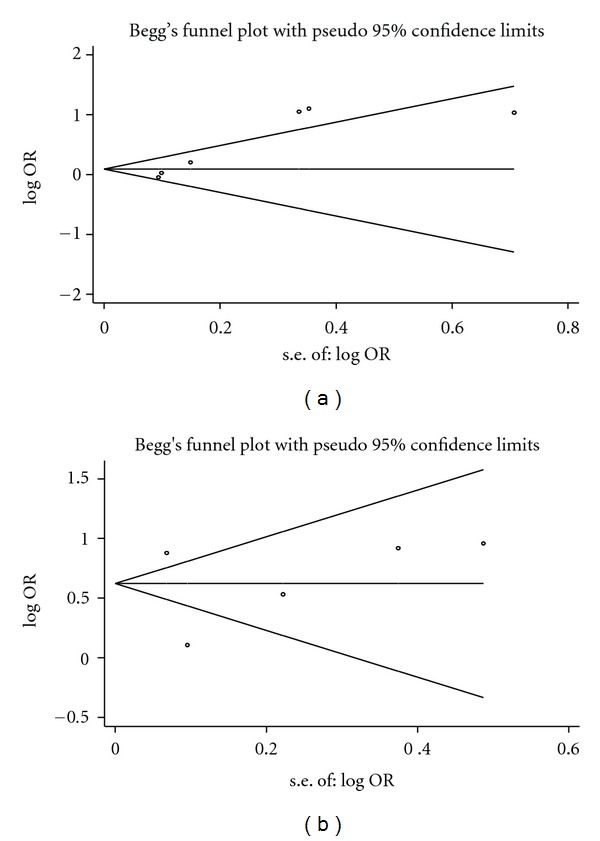
(a) Plot for arsenic concentration as exposure assessment, (b) funnel plot for arsenic concentration and duration as exposure assessment.

Investigation of the publication bias showed some evidence of positive results for the concentration and duration studies. The Egger's test *P* value for arsenic concentration studies was 1.00, while the Egger's test *P* value for the concentration and duration studies was 0.004. The funnel plot shows possible bias only for concentration and duration studies (Figure 3).

### 5.3. Metaregression

Using the scores from the *Quality* assessment, a metaregression was performed to verify the dependency of the *Outcome* measure on the quality scores. Of all the scoring criteria, the *Quality* of exposure measurement and total quality was significantly associated with the *Outcome* measure. For one unit increase in the score from the *Exposure* domain, the OR decreased by 6% (*P* = 0.004, 95% CI: 2.1%–10.7%).

## 6. Discussion

In the studies included in the meta-analysis, arsenic exposure was measured by different studies in different ways. Even when two studies used the same units of measurement, they may still have differed in categorization and definition of subjects being exposed to arsenic. The common units used were mg/L-year and *μ*g/L. However, the definition of “*exposure*” differs from one study to another, since there is no agreement on how to express the duration of exposure or what quantities of arsenic constitute an exposure. This problem results from the fact that external validation of the methods used to determine exposure in studies is still problematic. In addition, while methods for detection of exposure to high levels of arsenic have been well characterized, measurement of arsenic exposure at low concentrations is still not very reliable. Furthermore, Wang et al. [5] and Chen et al. [19] have followed the WHO protocol for the measurement of blood pressure and have used the criteria of diastolic blood pressure >95 mm Hg or systolic blood pressure >160 mm Hg to define hypertension, while Huang et al. [20], Rahman et al. [21], and Chen et al. [6] specified hypertension as diastolic > 90 mm Hg or systolic >140 mm Hg. In the study by Jones et al. [22], they followed diastolic blood pressure >90 mm Hg or systolic blood pressure >140 mm Hg to define hypertension.

Overall, this meta-analysis suggests that chronic arsenic exposure is likely to be associated with hypertension. The pooled OR of arsenic concentration studies is 1.9 (95% CI: 1.2–3.0) and that for arsenic concentration and duration studies is 1.4 (95% CI:.95–2.0). The *P* values for homogeneity for arsenic exposure were significant at *P* < 0.05. The I^2^ was 90% for arsenic concentration studies and 82.81% for arsenic concentration and duration studies. Investigation of publication bias showed some evidence of positive results for concentration and duration studies. The Egger's test *P* value for arsenic concentration studies was 0.828, while the *P* value for concentration and duration studies was 0.04.

Chen et al. [10] obtained similar results in their 2007 review of arsenic, diabetes, and hypertension. This paper included only four studies regarding arsenic exposure and hypertension and did not include a meta-analysis. In our meta-analysis, seven good quality studies were included that related arsenic exposure to hypertension using proper statistical analysis, with control groups (Table 1). As opposed to the report of Chen et al. [6], which only presented the results of their reviewed papers, the meta-analysis conducted here attempted to pool the reported ORs, tested for heterogeneity, and conducted a meta-regression. These are the major strengths of the current study. To the best of our knowledge, this is the first meta-analysis that has explored the association between hypertension and chronic arsenic exposure. One of the major limitations of this meta-analysis is the limited number of studies examining the association between hypertension and chronic arsenic exposure. These studies were also heterogeneous as reflected by the high values of the I^2^ statistics. Thus, a random effects model was used in conducting the meta-analysis.

The adverse effects of arsenic on blood vessels have been demonstrated in previous studies which reported that constant exposure of rats and rabbits to arsenite caused a considerable increase in peripheral vascular resistance.

The eight studies evaluated in this paper have shown how varied the impact of arsenic on hypertension, depending on factors such as sex, age, body-mass index, diabetes, and triglyceride levels of the subject population. However, an increased risk for hypertension among arsenic exposed participants has not been consistently observed. For instance, Dastgiri et al. [23] reported a significant difference both for systolic and diastolic blood pressures between exposed and unexposed subjects, while Khan et al. [24] reported no significant effect. Inconsistent findings may be due to differences in measurement criteria both for exposure and outcome variables, or due to bias caused by limited consideration of potential confounders. Due to these inconsistencies, the increased prevalence of hypertension for a population that is exposed to high arsenic levels cannot yet be established.

## 7. Conclusions and Recommendations

This meta-analysis suggests a possible association between chronic arsenic exposure and hypertension. However, the small number of studies and limitations in study quality pose challenges to establishing causation between arsenic exposure and hypertension. Nevertheless, since a larger proportion of the rural Bangladeshi population drink arsenic contaminated water and the risk of hypertension is increasing among the general population, even a small association may lead to a large number of cases and a large population attributable risk. Therefore, the association deserves further investigation, preferably with cohort studies.

## Figures and Tables

**Table 1:** Findings from hypertension and chronic exposure to arsenic studies.

Reference	Study design and sample size	Location and population	Exposure assessment	Outcome assessment	Variables adjusted for		Findings	
						Exposure categories	Prevalence	Odds ratios (95% CI)

Chen et al. [19]	Cross-sectional: 382 men and 516 women of age over 30 and residing in arseniasis hyperendemic villages of Taiwan Exposed: 779 (drunk artesian well for some time) Unexposed: 119 (never used artesian well)	Taiwan	Long-term arsenic exposure calculated, structured questionnaire, and measured arsenic concentration in well water Cumulative arsenic exposure index was used	WHO protocol: Systolic blood pressure ≥160 mm Hg, Diastolic blood pressure ≥95 mm Hg AND/OR history of hypertension regularly treated with antihypertensive drugs	Age, sex, body mass index, fasting serum triglyceride levels, diabetes mellitus, proteinuria	0 years 1–10 years 11–20 years 21–30 years >30 years 0 *μ*g/L 10–700 *μ*g/L 710–900 *μ*g/L >900 *μ*g/L Unknown 0 *μ*g/L-years 100–6300 *μ*g/L years 6400–10800 *μ*g/L-years 10900–14700 *μ*g/L-years 14800–18500 *μ*g/L-years >18500 *μ*g/L-years Unknown	Case: 5.0% Case: 3.8% Case: 15.4% Case: 24.7% Case: 27.7% Case: 5.0% Case: 20.0% Case: 28.8% Case: 19.2% Case: 17.0% Case: 5.0% Case: 4.9% Case: 12.8% Case: 22.1% Case: 26.5% Case: 29.2% Case: 17.0%	1.0 1.1 (0.2–4.6) 3.2 (1.2–8.2) 3.0 (1.2–7.3) 1.9 (0.7–6.0) 1.0 2.5 (1.0–6.2) 3.6 (1.4–9.1) 2.6 (1.0–6.9)* 1.7 (0.7–4.6) 1.0 0.9 (0.2–3.3) 2.4 (0.8–6.9) 3.6 (1.4–9.6) 3.6 (1.4–9.3) 2.8 (1.1–7.1)* 1.7 (0.7–4.6)

Rahman et al. [21]	Cross-sectional: 1595 residents 30 years or older Exposed: 1481 (had a history of arsenic-contaminated drinking water) Unexposed: 114 (had no history of arsenic-contaminated drinking water)	Bangladesh	Arsenic concentration measured in artesian well water and consumption was assessed by structured questionnaire to estimate time-weighted mean arsenic levels (mg/L) for each subject	Systolic blood pressure ≥140 mm Hg, AND diastolic blood pressure ≥90 mm Hg	Age, sex, body mass index	Unexposed <500 *μ*g/L 500–1000 *μ*g/L >1000 *μ*g/L Unexposed <1000 *μ*g/L 1000–5000 *μ*g/L 5000–10000 *μ*g/L >10000 *μ*g/L	Case: 7.9% Case: 8.0% Case: 16.1% Case: 19.5% Case: 7.9% Case: 5.5% Case: 12.0% Case: 14.3% Case: 22.9%	1.0 1.2 (0.6–2.3) 2.2 (1.1–4.3) 2.5 (1.2–4.9)* 1.0 0.8 (0.3–1.7) 1.5 (0.7–2.9) 2.2 (1.1–4.4) 3.0 (1.5–5.8)* Dose response among exposed *P* value <0.001

Zieroldet al. [18]	Cross-sectional: 1185 residents from Wisconsin, USA, who are 35 years or older and were drinking from private well water supply for 20 or more years Exposed: 40 (>2 *μ*g/L) Unexposed: 34 (<2 *μ*g/L)	Wisconsin, USA	Questionnaire survey for life-time history, usual drinking water consumption, use of water-treatment systems and family health history. Length of exposure estimated between July 2000 and January 2002	Self-reported	Gender, age, smoking status, body mass index	<2 *μ*g/L 2 *μ*g/L–10 *μ*g/L >10 *μ*g/L		1.0 1.2 (0.8–1.6) 1.7 (1.1–2.5)*

Wang et al. [5]	Prospective cohort: 18940 residents 40 years or older followed up for an average of 12 years Exposed: 2264 (residents from arsenic-exposed areas and drinking from tube wells) Unexposed: 16676 (residents from unexposed areas and drinking from tap water)	Taiwan	In one area, median arsenic concentration of the shared artesian well was used as exposure, whereas, in other area, the exposure was the arsenic level in the participants' own tube wells. People using tap water or drinking water with <10 *μ*g/L of arsenic were considered unexposed.	Systolic blood pressure ≥160 mm Hg, diastolic blood pressure ≥95 mm Hg, OR history of hypertension regularly treated with antihypertensive agents	Age, gender	<10 *μ*g/L 10–49 *μ*g/L 50–499 *μ*g/L >500 *μ*g/L —	Case: 8.1% Case: 22.0%	1.0 2.4 (2.1–2.7)*

Chen [25]	Cross-sectional: 11,458 men and women from Bangladesh who are 18 years or older	Bangladesh		Systolic blood pressure ≥140 mm Hg Diastolic blood pressure ≥90 mm Hg Systolic blood pressure ≥140 mm Hg AND/OR Diastolic blood pressure ≥90 mm Hg Pulse pressure ≥55 mm Hg	Age, gender, body mass index, cigarette smoking status, education length	0.1–7.0 7.3–39.0 40.0–91.0 92.0–179.0 180.0–864.0 0.1–7.0 7.3–39.0 40.0–91.0 92.0–179.0 180.0–864.0 0.1–7.0 7.3–39.0 40.0–91.0 92.0–179.0 180.0–864.0 0.1–7.0 7.3–39.0 40.0–91.0 92.0–179.0 180.0–864.0 <48.1 48.1–227.8 227.9–585.8 585.9–1494.0 >1494.1 <48.1 48.1–227.8 227.9–585.8 585.9–1494.0 >1494.1 <48.1 48.1–227.8 227.9–585.8 585.9–1494.0 >1494.1 <48.1 48.1–227.8 227.9–585.8	Case: 6.7% Case: 9.3% Case: 7.7% Case: 8.1% Case: 7.7% Case: 9.1% Case: 9.9% Case: 8.9% Case: 8.7% Case: 8.8% Case: 11.9% Case: 13.8% Case: 12.0% Case: 12.1% Case: 12.2% Case: 9.0% Case: 11.4% Case: 11.0% Case: 10.4% Case: 10.6% Case: 7.2% Case: 8.2% Case: 8.1% Case: 8.3% Case: 8.0% Case: 10.2% Case: 8.5% Case: 9.1% Case: 9.0% Case: 8.9% Case: 13.2% Case: 11.9% Case: 12.3% Case: 12.8% Case: 12.3% Case: 9.1% Case: 11.6% Case: 10.8%	1.0 1.50 (1.20–1.88) 1.24 (0.99–1.57) 1.32 (1.05–1.66) 1.25 (0.99–1.57) 1.0 1.15 (0.94–1.41) 1.05 (0.85–1.29) 1.02 (0.83–1.26) 1.07 (0.87–1.31) 1.0 1.24 (1.04–1.49) 1.07 (0.89–1.29) 1.08 (0.90–1.30) 1.11 (0.92–1.33)* 1.00 1.33 (1.10–1.62) 1.27 (1.04–1.55) 1.19 (0.98–1.45) 1.20 (0.99–1.47) 1.00 1.19 (0.95–1.50) 1.28 (1.01–1.61) 1.26 (1.00–1.58) 1.15 (0.92–1.45) 1.00 0.84 (0.68–1.04) 0.96 (0.78–1.18) 0.93 (0.75–1.14) 0.91 (0.74–1.14) 1.00 0.91 (0.75–1.09) 0.99 (0.82–1.19) 1.01 (0.84–1.21) 0.95 (0.79–1.14)* 1.00 1.32 (1.10–1.62) 1.27 (1.04–1.55)

Chen [25]	Cross-sectional: 11,458 men and women from Bangladesh who are 18 years or older	Bangladesh		Systolic blood pressure ≥140 mm Hg Diastolic blood pressure ≥90 mm Hg Systolic blood pressure ≥140 mm Hg and/OR Diastolic blood pressure ≥90 mm Hg Pulse pressure ≥55 mm Hg	Age, gender, body mass index, cigarette smoking status, education length	585.9–1494.0 >1494.1 ≤90.0 90.1–159.2 159.3–245.9 246.0–405.4 >405.4 ≤90.0 90.1–159.2 159.3–245.9 246.0–405.4 >405.4 ≤90.0 90.1–159.2 159.3–245.9 246.0–405.4 >405.4 ≤90.0 90.1–159.2 159.3–245.9 246.0–405.4 >405.4	Case: 11.2% Case: 10.1% Case: 8.3% Case: 8.6% Case: 8.0% Case: 7.5% Case: 6.6% Case: 10.7% Case: 9.9% Case: 9.4% Case: 7.7% Case: 7.7% Case: 14.0% Case: 13.6% Case: 12.4% Case: 11.1% Case: 10.6% Case: 10.1% Case: 10.7% Case: 10.9% Case: 10.1% Case: 10.1%	1.19 (0.98–1.45) 1.20 (0.99–1.47) 1.00 1.14 (0.91–1.42) 1.12 (0.90–1.41) 1.11 (0.89–1.40) 1.09 (0.86–1.39) 1.00 1.00 (0.82–1.23) 1.06 (0.86–1.30) 0.87 (0.70–1.08) 0.99 (0.80–1.24) 1.00 1.04 (0.87–1.24) 1.02 (0.85–1.22) 0.93 (0.77–1.12) 0.99 (0.82–1.20) 1.00 1.07 (0.88–1.31) 1.10 (0.91–1.34) 1.05 (0.86–1.58) 1.09 (0.89–1.34)

Huang et al. [20]	Cross-sectional: 488 women and 383 men southwestern coast of Taiwan whose age is 30 years or older	Taiwan	Percentage of inorganic arsenic in urine was used to determine exposure	WHO protocol: Systolic blood pressure ≥140 mm Hg, Diastolic blood pressure ≥90 mm Hg, AND/OR history of hypertension regularly treated with antihypertensive drugs	Age, gender, body mass index, cigarette smoke, alcohol consumption, triglyceride level, cumulative arsenic exposure	*InAs% <4.53% 4.53%–8.00% ≥8.00% CAE = 0 *μ*g/L-year InAs%** <61000 ≥61000 CAE > 0 *μ*g/L InAs% <61000% ≥61000	Case: 37.3% Case: 30.6% Case: 41.2% Case: 21.3% Case: 22.0% Case: 38.6% Case: 56.5%	1.0 0.72 (0.46–1.11) 1.21 (0.79–1.85) *P* for trend *P*-value: 0.35 1.0 1.04 (0.43–2.48) 2.31 (1.19–4.48)* 2.84 (1.47–5.46)* * *P* < 0.05 **P < 0.01

Chen et al. [6]	Cross-sectional: 10,910 participants from Bangladesh who are married, 18 years or older, and have had resided in the area for at least 5 years	Bangladesh	Arsenic concentration was measured in the wells to estimate time-weighted well arsenic concentration (TWA) combining the water use behaviour for, an average, last 10 years for men and 8.3 years for women	Overall population: Systolic hypertension ≥140 mm Hg Diastolic blood pressure ≥90 mm Hg Systolic hypertension ≥140 mm Hg AND/OR Diastolic blood pressure ≥90 mm Hg Pulse blood pressure ≥55 mm Hg	Age, gender, body mass index, cigarette smoking status, education length, daily water consumption	(Mean years exposed) 0.1 *μ*g/L–8.0 *μ*g/L (2.8 years) 8.1 *μ*g/L–40.8 *μ*g/L (23.2 years) 40.9 *μ*g/L–91.0 *μ*g/L (63.9 years) 91.1 *μ*g/L–176.0 *μ*g/L (128.1 years) 176.1 *μ*g/L–864.0 *μ*g/L (283.1 years) 0.1 *μ*g/L–8.0 *μ*g/L (2.8 years) 8.1 *μ*g/L–40.8 *μ*g/L (23.2 years) 40.9 *μ*g/L–91.0 *μ*g/L (63.9 years) 91.1 *μ*g/L–176.0 *μ*g/L (128.1 years) 176.1 *μ*g/L–864.0 *μ*g/L (283.1 years) 0.1 *μ*g/L–8.0 *μ*g/L (2.8 years) 8.1 *μ*g/L–40.8 *μ*g/L (23.2 years) 40.9 *μ*g/L–91.0 *μ*g/L (63.9 years) 91.1 *μ*g/L–176.0 *μ*g/L (128.1 years) 176.1 *μ*g/L–864.0 *μ*g/L (283.1 years) 0.1 *μ*g/L–8.0 *μ*g/L (2.8 years) 8.1 *μ*g/L–40.8 *μ*g/L (23.2 years) 40.9 *μ*g/L–91.0 *μ*g/L (63.9 years) 91.1 *μ*g/L–176.0 *μ*g/L (128.1 years) 176.1 *μ*g/L–864.0 *μ*g/L (283.1 years)	Case: 7.3% Case: 8.9% Case: 8.0% Case: 8.2% Case: 7.6% Case: 10.0% Case: 8.9% Case: 9.4% Case: 8.5% Case: 8.8% Case: 12.9% Case: 12.9% Case: 12.5% Case: 11.9% Case: 12.1% Case: 9.1% Case: 11.9% Case: 10.6% Case: 10.4% Case: 10.7%	1.00 1.39 (1.10–1.75) 1.21 (0.96–1.54) 1.28 (1.01–1.62) 1.13 (0.90–1.44) 1.00 0.96 (0.77–1.2) 1.01 (0.81–1.25) 0.93 (0.75–1.16) 0.97 (0.78–1.2) 1.00 1.1 (0.90–1.33) 1.03 (0.85–1.25) 1.01 (0.83–1.22) 1.02 (0.84–1.23)* 1.00 1.39 (1.14–1.71) 1.21 (0.99–1.49) 1.19 (0.97–1.45) 1.19 (0.97–1.46)

Chen et al. [6]	Cross-sectional: 10,910 participants from Bangladesh who are married, 18 years or older, and have had resided in the area for at least 5 years	Bangladesh		Subpopulation (≥5 years exposure): Systolic hypertension ≥140 mm Hg Diastolic blood pressure ≥90 mm Hg Systolic hypertension ≥140 mm Hg AND/OR Diastolic blood pressure ≥90 mm Hg Pulse blood pressure ≥55 mm Hg	Age, gender, body mass index, cigarette smoking status, education length, daily water consumption	(Mean years exposed) 0.1 *μ*g/L–8.0 *μ*g/L (2.7 years) 8.1 *μ*g/L–40.8 *μ*g/L (23.2 years) 40.9 *μ*g/L–91.0 *μ*g/L (64.3 years) 91.1 *μ*g/L–176.0 *μ*g/L (127.2 years) 176.1 *μ*g/L–864.0 *μ*g/L (284.7 years) 0.1 *μ*g/L–8.0 *μ*g/L (2.7 years) 8.1 *μ*g/L–40.8 *μ*g/L (23.2 years) 40.9 *μ*g/L–91.0 *μ*g/L (64.3 years) 91.1 *μ*g/L–176.0 *μ*g/L (127.2 years) 176.1 *μ*g/L–864.0 *μ*g/L (284.7 years) 0.1 *μ*g/L–8.0 *μ*g/L (2.7 years) 8.1 *μ*g/L–40.8 *μ*g/L (23.2 years) 40.9 *μ*g/L–91.0 *μ*g/L (64.3 years) 91.1 *μ*g/L–176.0 *μ*g/L (127.2 years) 176.1 *μ*g/L–864.0 *μ*g/L (284.7 years) 0.1 *μ*g/L–8.0 *μ*g/L (2.7 years) 8.1 *μ*g/L–40.8 *μ*g/L (23.2 years) 40.9 *μ*g/L–91.0 *μ*g/L (64.3 years) 91.1 *μ*g/L–176.0 *μ*g/L (127.2 years) 176.1 *μ*g/L–864.0 *μ*g/L (284.7 years)	Case: 7.6% Case: 9.0% Case: 8.9% Case: 8.4% Case: 7.7% Case: 10.2% Case: 8.8% Case: 10.2% Case: 8.4% Case: 9.1% Case: 12.9% Case: 12.6% Case: 13.7% Case: 12.0% Case: 12.5% Case: 8.7% Case: 11.9% Case: 11.1% Case: 10.9% Case: 10.5%	1.35 (1.02–1.77) 1.28 (0.97–1.69) 1.3 (0.99–1.72) 1.12 (0.85–1.47) 1.00 0.94 (0.72–1.22) 1.07 (0.83–1.38) 0.93 (0.72–1.20) 1.00 (0.78–1.28) 1.00 1.06 (0.84–1.34) 1.12 (0.89–1.41) 1.03 (0.82–1.30) 1.05 (0.84–1.31) 1.00 1.5 (1.16–1.91) 1.34 (1.04–1.73) 1.35 (1.05–.71) 1.24 (0.97–1.59)

Jones et al. [22]	Prospective cohort: 4167 adults (men) of age 20 years or older who participated in the survey from 2003 to 2008	USA	Total arsenic concentration (*μ*g/L) in urine was used as exposure level	Overall population: Systolic hypertension ≥140 mm Hg Diastolic blood pressure ≥90 mm Hg	Sex, age, race/ethnicity, education, BMI, smoking status, continine, serum folate, vitamin12, diabetes, and alcohol consumption	Total arsenic (*μ*g/L) <4.2	4.2–8.3 >8.3–17.1	>17.1
						Model 2 1.03 (0.96–1.10)	1.001.09 (0.84–1.41)	1.33 (0.99–1.80) 1.22 (0.91–1.64)*
						Total arsenic minus arsenobetaine ((*μ*g/L)		
							<3.13.1–5.8	>5.8–10.8>10.8
						Model 21.05 (0.96–1.14)	1.001.07 (0.81–1.42)	1.34 (0.95–1.89) 1.30 (092–1.83)
						Dimethylarsinate ((*μ*g/L)		
							<2.02.0–3.6	>3.6–6.0>6.0
						Model 21.12 (1.01–1.23)	1.001.06 (0.80–1.41)	1.21 (0.89–1.64) 1.29 (0.93–1.79)

*OR included in the meta-analysis.

**PMI: primary methylation index, InAs.

**Table 2:** Average quality scores on quality scores for studies included in the meta-analysis.

Categories of quality scoring (maximum points value)	Average (%) of the maximum category quality score (range)		ICC^a^ for interobserver agreement (95% CI)
	Cross-sectional (*n* = 7)	Cohort studies (*n* = 1)	

Selection (20 points)	15.8 (14.7–16.0)	18.7	0.659 (−0.015–0.922)
Exposure (30 points)	24.3 (18.3–30.0)	20.0	0.870 (0.563–0.972)
Outcome (20 points)	14.7 (1.3–17.3)	16.0	0.958 (0.644–0.992)
Confounders (20 points)	18.6 (13.3–20.0)	10.0	0.578 (−0.188–0.902)
Analysis (10 points)	10 (10.0-10.0)	9.3	0.001 (−0.238–0.782)
Total quality score (100 points)	84.1 (61.0–93.3)	74.0	0.884 (0.620–0.975)

^a^ICC: intraclass correlation coefficient.
